# Protective effect of ginsenoside Rg1 on lidocaine-induced apoptosis

**DOI:** 10.3892/mmr.2013.1822

**Published:** 2013-11-21

**Authors:** HUI LI, JUNMEI XU, XIN WANG, GUIXIU YUAN

**Affiliations:** Department of Anesthesiology, Second Xiangya Hospital, Central South University, Changsha, Hunan 410011, P.R. China

**Keywords:** Rg1, lidocaine, caspase-3, B-cell lymphoma-2, apoptosis, anesthesia

## Abstract

Lidocaine, as an anesthetic substance, is often used for surface and spinal anesthesia. However, studies have shown that lidocaine may induce transient neurological symptoms and cauda equina syndrome. In the present study the effects of the ginsenoside Rg1 (Rg1) on lidocaine-induced apoptosis were assessed in Jurkat cells using flow cytometry and terminal deoxynucleotidyl transferase-mediated dUTP nick end labeling (TUNEL). The data showed that incubation with Rg1 provides protection against lidocaine-induced apoptosis in cultured Jurkat cells. In order to investigate the effect of Rg1 on the apoptosis pathway, caspase 3 gene expression was determined. The results suggested that the protective effect of Rg1 on lidocaine-induced apoptosis is mediated by altering the level of B-cell lymphoma-2 (BCL-2) family proteins and downregulating caspase-3 expression. In conclusion, the present study demonstrated that incubation with Rg1 provides protection against lidocaine-induced apoptosis in cultured Jurkat cells. In addition, the study demonstrated that Rg1 is a notable antiapoptotic molecule that is capable of blocking the caspase-dependent signaling cascade in Jurkat cells, and that the protective effect of Rg1 on lidocaine-induced apoptosis is mediated by altering levels of BCL-2 family proteins and downregulating caspase-3 expression. The present study provides the basis for understanding and evaluating the effect of Rg1 in the *in vivo* treatment of lidocaine-induced transient neurological symptoms and cauda equina syndrome by lidocaine.

## Introduction

Lidocaine, which is a common local anesthetic and antiarrhythmic drug, may lead to transient neurological symptoms and cauda equina syndrome following spinal anesthesia (1). Despite this, spinal anesthesia with lidocaine is a relatively widely used technique. Several studies have demonstrated that the cytotoxicity of lidocaine is dose- and time-dependent in rats and mice (2,3), and it has been shown *in vitro* that lidocaine induces apoptosis (4). This apoptotis-inducing effect is due to mitochondrial injury (5). Apoptosis is controlled by caspases, which are activated by two major signaling pathways, the extrinsic death receptor and intrinsic mitochondrial pathways. Werdehausen *et al* (6) reported that the apoptosis induced by lidocaine is triggered by the intrinsic mitochondrial death pathway rather than by death receptors. Therefore, B-cell lymphoma-2 (BCL-2) and caspase-3 are useful indicators of lidocaine-induced apoptosis.

Ginsenosides are found in the popular herb *Panax ginseng*, which is widely used in traditional Chinese medicine (7). Ginseng has been demonstrated to exert pharmacological effects on the central nervous, cardiovascular, endocrine and immune systems (8). For a long time the curative effects of ginseng were attributed to the mixed results from *Panax ginseng* rather than to the individual biologically active compounds. However, several studies have since identified ginsenosides, including Rb, Rc, Re, Rg and Rh, as the key ingredient for the pharmacological actions (9,10). Among these ginsenosides, ginsenoside Rg1 (Rg1) is considered to be one of the most active and abundant steroid saponins, and acts as an antioxidant (11). Rg1 is crucial in the modulation of neurotransmission and the prevention of scopolamine-induced memory deficits, and acts by increasing cholinergic activity (12). Rg1 has also been demonstrated to increase humoral and cell-mediated immune responses (13).

To date, lidocaine-induced cytotoxicity, which may result in transient neurological symptoms and cauda equina syndrome, has been widely accepted (14,15). Thus, a novel strategy is required to protect against the cytotoxicity of lidocaine. In the present study the effect of Rg1 on lidocaine-induced apoptosis was assessed in human Jurkat T-lymphoma cells. In addition, the expression levels of the regulators of lidcaine-induced apoptosis, including BCL-2 and caspase-3, were examined. It was hypothesized that Rg1 is a potential drug for lidocaine-induced cytotoxicity. The present study may provide novel insights into therapeutic options for transient neurological symptoms and cauda equina syndrome following spinal anesthesia.

## Materials and methods

### Cell culture and treatment

An acute human T lymphoma Jurkat cell line was provided by the Laboratory of Molecular Biology, Xiangya Medical College of Central South University (Changsha, China). The cells were maintained in RPMI-1640 medium (Life Technologies, Inc., Carlsbad, CA, USA) supplemented with 10% fetal bovine serum (Life Technologies, Inc.), 50 U/ml penicillin, 50 μg/ml streptomycin and 2 mM glutamine at 37°C in a humidified incubator containing 5% CO_2_. Having been cultured for five days, the cells were seeded in a 96-well microtiter plate at a density of 2×10^4^ cells/well. The cells were divided into five groups: Control, 3 mM lidocaine without Rg1 pretreatment, 6 mM lidocaine without Rg1 pretreatment, 3 mM lidocaine with Rg1 pretreatment and 6 mM lidocaine with Rg1 pretreatment. The 3 mM lidocaine with Rg1 pretreatment and 6 mM lidocaine with Rg1 pretreatment groups were incubated with 50 mg/l Rg1 for 2 h prior to lidocaine treatment. All groups, except the control group, were then incubated with the corresponding lidocaine concentration for 16 h, ready for further assays.

### Apoptosis analysis using flow cytometry and terminal deoxynucleotidyl transferase-mediated dUTP nick end labeling (TUNEL) assay

To investigate the effect of Rg1 on cell apoptosis in Jurkat cells stimulated by lidocaine, a flow cytometry assay was performed. All fluorescence signals of labeled cells were analyzed using the FACScan™ flow cytometer (Becton-Dickinson, San Jose, CA, USA). The measurement of phosphatidylserine redistribution on the plasma membrane was conducted using an Annexin V-fluorescein isothiocyanate (FITC) Apoptosis Detection kit (Becton Dickinson) according to the manufacturer’s instructions.

DNA fragmentation was evaluated using a fluorescein-TUNEL assay with an Apo-Direct kit (BD, Pharmingen, San Diego, CA, USA) according to the manufacturer’s instructions. Positive and negative controls provided by the manufacturer and internal controls (specimens with known DNA damage) were included for each run. Following washing in phosphate-buffered saline (PBS) to remove ethanol, the cell pellets were resuspended in 50 μl freshly prepared staining solution for 60 min at 37°C. The staining solution contained terminal deoxytransferase (TdT) enzyme, TdT reaction buffer, FITC-tagged dUTP nucleotides and distilled water.

### Quantitative polymerase chain reaction (PCR)

Quantitative PCR was used to measure the RNA transcripts. Total RNA was isolated from cultured cells of different treatment groups using TRIzol reagent (Invitrogen Life Technologies, Carlsbad, CA, USA) according to the manufacturer’s instructions. Total RNA (1 μg) was digested with DNase I (Fermentas, Vilnius, Lithuania) to eliminate residual DNA and transcribed to cDNA with oligo (dT)_12–18_ primer using a reverse transcription system (First-Strand cDNA kit; Fermentas). The single-stranded cDNA was amplified by quantitative PCR with BCL-2 and caspase-3 primer pairs in a Prism 7500 Sequence Detection system (Applied Biosystems, Foster City, CA, USA) (Table I). β-actin was used as an endogenous control (Table I). The reaction conditions were as follows: 50°C for 5 min and 95°C for 10 min followed by 40 cycles at 95°C for 15 sec and 60°C for 45 sec. Dissociation curves were then used to examine whether the PCR products were specific. The results were corrected according to PCR efficiency, tested for significance by a pair-wise fixed reallocation randomization test (iterations=10,000) and plotted using standard error estimation via a complex Taylor algorithm.

### Western blot analysis

Following incubation, the cells of each group were collected by centrifugation at 200 × g for 5 min (Eppendorf, Hamburg, Germany) at 4°C and washed three times with ice-cold PBS, pH 7.4, prior to undergoing centrifugation at 200 × g for 5 min. The cell pellets were resuspended in radioimmunoprecipitation assay (RIPA) lysis buffer (Pierce, Rockford, IL, USA). To obtain the solubilized cellular proteins, lysates were centrifuged at 10,000 × g for 15 min at 4°C. Proteins were separated using 15% polyacrylammide gels and elecrotransferred to polyvinylidene diflouride membranes. The membranes were blocked and incubated with BCL-2 (monoclonal, mouse, ab692; Abcam, Cambridge, MA, USA) and caspase-3 (monoclonal, mouse, ab2171; Abcam) antibodies, followed by horseradish peroxidase-conjugated second antibody (monoclonal, rabbit, ab97046; Abcam). Subsequent to washing with PBS three times, the signals were detected by Enhanced Chemiluminescence (ECL) Reagent (Amersham, Little Chalfont, UK). For an internal control, β-actin was used to normalize the target bands.

### Immunofluorescence

Human T lymphoma Jurkat cells were grown on 24×24 mm cover glasses and fixed for 30 min in 4% paraformaldehyde solution in phosphate buffer, prior to 30 min incubation with blocking reagent (5% fetal bovine serum in PBS). Primary antibodies of BCL-2 and caspase-3 were incubated for ≥1 h at room temperature prior to washing. Immunofluorescence staining was performed with secondary antibodies and 4′,6-diamidino-2-phenylindole. A conventional fluorescence microscope (Axioskop 20;Carl Zeiss Inc., Thornwood, NY, USA) was used for visualization.

### Statistical analysis

Data are expressed as the mean ± standard deviation (SD). Statistical analysis of the data was performed using the Duncan’s test. P<0.05 was considered to indicate a statistically significant difference.

## Results

### Protective effect of Rg1 on lidocaine-induced apoptosis

**Figure 1:**
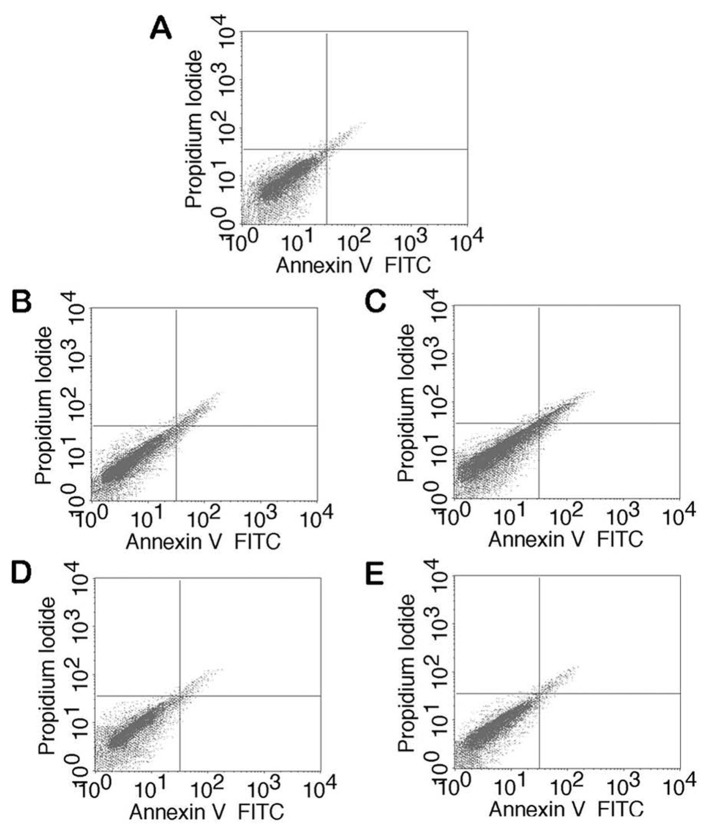
Flow cytometry assay of (A) the control group, (B) the 3 mM lidocaine without ginsenoside Rg1 (Rg1) pretreatment group, (C) the 6 mM lidocaine without Rg1 pretreatment group, (D) the 3 mM lidocaine with Rg1 pretreatment group and (E) the 6 mM lidocaine with Rg1 pretreatment group. FITC, fluorescein isothiocyanate.

The effect of Rg1 on lidocaine-induced apoptosis was determined using flow cytometry and TUNEL assays. The results from the flow cytometry assay revealed that lidocaine significantly increased the number of early- [Annexin V^+^/propidium iodide (PI^−^)] and late-stage (Annexin V^+^/PI^+^) apoptotic bone marrow stromal cells (BMSCs) compared with the negative control group. The Rg1-pretreated groups, however, exhibited decreased lidocaine-induced apoptosis in BMSCs, including early- and late-stage apoptosis, compared with the groups without Rg1 pretreatment (Fig. 1).

**Figure 2:**
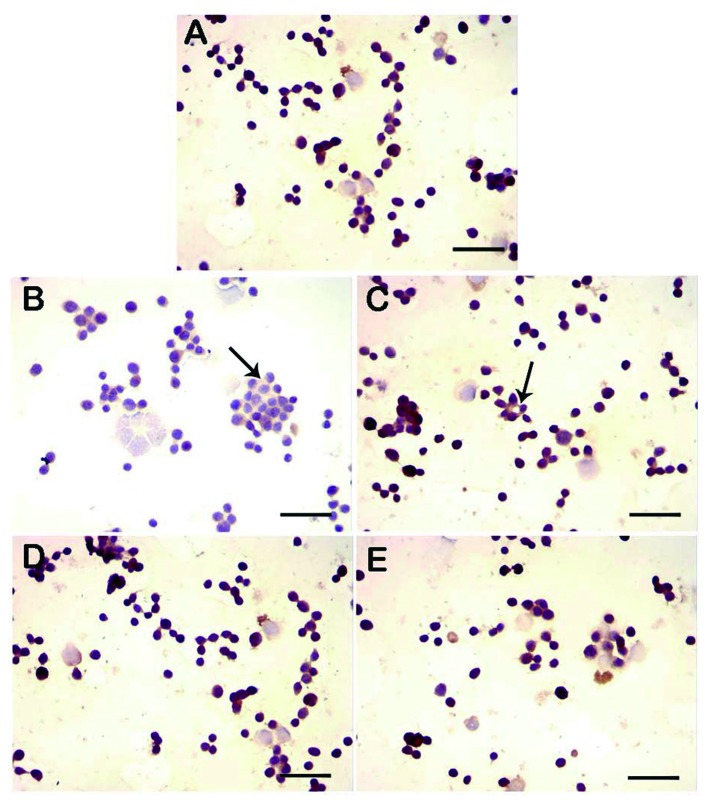
Terminal deoxynucleotidyl transferase-mediated dUTP nick end labeling (TUNEL) assay of (A) the control group, (B) the 3 mM lidocaine without ginsenoside Rg1 (Rg1) pretreatment group, (C) the 6 mM lidocaine without Rg1 pretreatment group, (D) the 3 mM lidocaine with Rg1 pretreatment group and (E) the 6 mM lidocaine with Rg1 pretreatment group. The arrows indicate positive signals of apoptosis. Scale bar, 50 μm.

DNA fragmentation was assessed in cultures from the control, lidocaine-treated and lidocaine with Rg1 pretreatment groups using the TUNEL assay. Compared with the control group, lidocaine significantly increased apoptosis in Jurkat cells. The lidocaine with Rg1 pretreatment groups, however, showed intermediary apoptosis compared with the control and lidocaine groups (Fig. 2).

### Rg1 suppresses the expression of caspase-3

**Figure 3:**
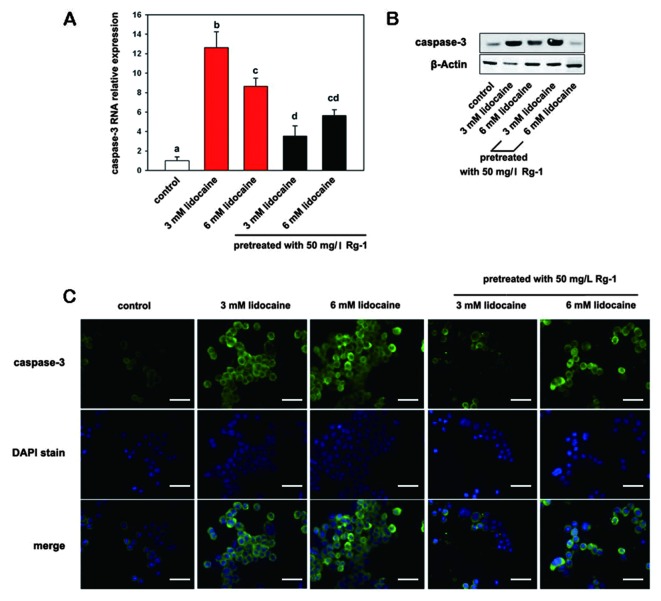
Caspase-3 expression of the control, 3 mM lidocaine without ginsenoside Rg1 (Rg1) pretreatment, 6 mM lidocaine without Rg1 pretreatment, 3 mM lidocaine with Rg1 pretreatment and 6 mM lidocaine with Rg1 pretreatment groups. (A) Relative mRNA expression of the studied groups. Different characters indicate significant difference (P<0.05). (B) Western blot analysis of the studied groups. (C) Immunofluorescence assay of the studied groups. Green signal, green fluorescent protein; blue signal, DAPI. Scale bar, 50 μm. DAPI, 4′,6-diamidino-2-pheylindole.

To assess the antiapoptotic effect of Rg1, quantitative PCR was performed using specific primers for caspase-3. In addition, protein expression of caspase-3 was detected using western blot analysis and immunofluorescence. The results from the RNA assay showed that lidocaine significantly induced caspase-3 expression (P>0.05); however, following pretreatment with Rg1 the induced caspase-3 expression was significantly decreased (P≤0.05) (Fig. 3A). Similarly, there was a significant increase in caspase-3 protein expression following treatment with lidocaine, while pretreatment with Rg1 decreased lidocaine-induced caspase-3 protein expression (Fig. 3B). Caspase expression appears high in the 3 mM lidocain with Rg1 pretreatment group in Fig. 3B due to the reduced cellular apoptosis. Immunofluorescence showed strong, punctuate staining for caspase-3 in lidocaine-treated groups compared with the control and Rg1-pretreated groups (Fig. 3C).

### Rg1 increases the expression of BCL-2

**Figure 4:**
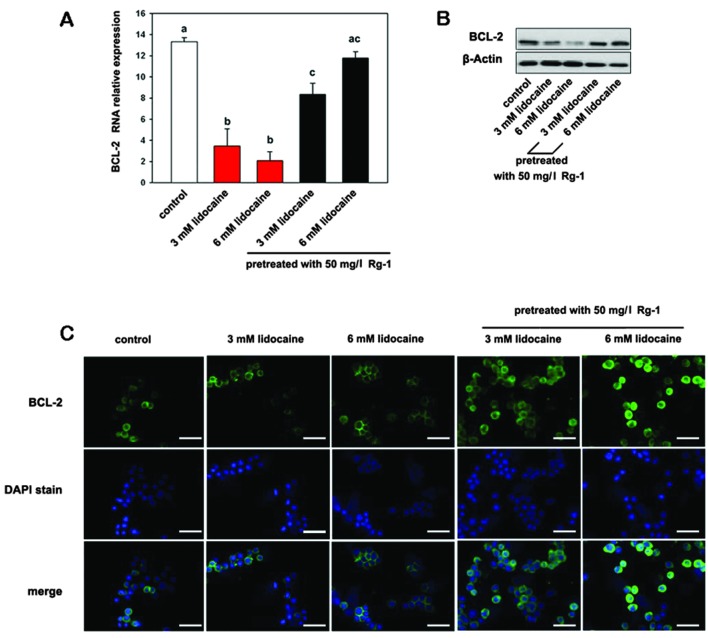
BCL-2 expression of the control group, the 3 mM lidocaine without ginsenoside Rg1 (Rg1) pretreatment group, the 6 mM lidocaine without Rg1 pretreatment group, the 3 mM lidocaine with Rg1 pretreatment group and the 6 mM lidocaine with Rg1 pretreatment group. (A) mRNA relative expression of the studied groups. Different characters suggested significant difference (P<0.05). (B) Western blot analysis of the studied groups. (C) Immunofluorescence assay of the studied groups. Green signal, green fluorescent protein; blue signal, DAPI. Scale bar, 50 μm. BCL-2, B-cell lymphoma-2; DAPI, 4′,6-diamidino-2-pheylindole.

The expression of BCL-2 mRNA and protein was decreased in Jurkat cells cultivated with lidocaine compared with the control cells. However, cells pretreated with Rg1 showed significantly higher BCL-2 mRNA and protein expression levels (Fig. 4). Rg1 thus exerts a cytoprotective effect against lidocaine-induced apoptosis in cultured Jurkat cells. Immunofluorescence showed strong, punctuate staining for BCL-2 in control and Rg1 pretreatment groups, compared with the groups treated only with lidocaine.

## Discussion

Lidocaine, as an anti-inflammatory, anesthetic and antiarrhythmic substance, is often used for surface and spinal anesthesia (16). However, studies have shown that lidocaine is capable of inducing transient neurological symptoms and cauda equina syndrome (17–19). It has been suggested that lidocaine promotes apoptosis in T-lymphoma cells, gingival fibroblasts, human chondrocytes and leukemia cells (6), and that lidocaine induces apoptosis intrathecally in a dose-dependent manner (5). This effect may be inhibited by overexpression of the cellular antiapoptotic protein BCL-2 or by caspase-9 deficiency (6). These results indicate that lidocaine may be dangerous for patients; however, lidocaine is still widely used for short-lasting regional anesthesia.

Ginsenoside Rg1 is a steroidal saponin abundantly found in ginseng (20). Ginsenosides are the most important active compounds identified in all species of ginseng. There are two predominant classes of ginsenosides, which are derived either from protopanaxatriol (Rg1, Rg2, Re and Rf) or protopanaxadiol (Rb1, Rb2, Rc and Rd) (21). In the present study, the effects of Rg1 on lidocaine-induced apoptosis were assessed in Jurkat cells using flow cytometry and TUNEL. The results showed that Rg1 pretreatment may protect Jurkat cells from lidocaine-induced apoptosis, presumably by acting as a biological antiapoptotic substance. Therefore, Rg1 may promote the recovery of Jurkat cells under similar pathological conditions.

To understand how the apoptosis pathway changes in response to Rg1, caspase-3 gene expression was examined. Caspases are cystein proteases that have a key role in the execution phase of apoptosis (22–24). Among the caspase family, caspase-3 has been widely studied, and has been proposed to have a crucial role in the cell death process (25,26). It has been shown that caspase-3 induces apoptosis through several different pathways, including degrading antiapoptotic proteins, and cleaving DNA repair molecules, extracellular matrix proteins, cytoskeletal proteins and other associated molecules (27). The results of the present study showed that expression of caspase-3 increased when Jurkat cells were treated with lidocaine, and that this was positively correlated with the rate of apoptosis. However, pretreatment with Rg1 decreased the expression of caspase-3 and the rate of apoptosis. These results suggested that apoptosis of Jurkat cells induced by lidocaine is associated with the upregulation of the expression of caspase-3, and that Rg1 reduces the rate of apoptotisis by downregulating the expression of caspase-3. In this study, lidocaine induced Jurkat cell apoptosis, as indicated by increased DNA fragmentation and caspase-3 activation. However, these apoptotic events were blocked by Rg1, indicating that Rg1 inhibits the mitochondrial apoptotic signaling pathway.

It has been shown that the release of proapoptotic proteins from mitochondria is modulated by targeting BCL-2 family members to the outer mitochondrial membrane (28). The BCL-2 family of proteins, which consists of anti- and pro-apoptotic molecules, controls mitochondrial function in apoptosis and constitutes a critical intracellular checkpoint for apoptosis within a common cell death pathway (29,30). The BCL-2 family includes proteins that predispose cells to apoptosis, such as Bax and Bad, and proteins that antagonize apoptosis, such as BCL-2 (31). The balance between these proteins is important for the release of mitochondrial proapoptotic proteins. BCL-2 serves as a critical regulator of pathways involved in apoptosis, acting to inhibit cell death (32). Furthermore, the BCL-2 gene was revealed to be upregulated in failing, as well as in aging, hearts (33). The BCL-2 gene acts to prevent programmed cell death of ventricular myocytes (34). Therefore, in the present study, the effect of Rg1 on the expression of BCL-2 family genes was investigated in Jurkat cells. Quantitative PCR and western blot analysis revealed that lidocaine significantly increased the expression of caspase-3, an apoptosis promoting BCL-2 family member, and decreased antiapoptotic BCL-2 expression in Jurkat cells. In combination, these data suggest that Rg1 is able to block the mitochondrial apoptotic signaling pathway in Jurkat cells by modulating the expression and activity of BCL-2 family proteins. The identification of direct molecular targets of Rg1 that regulate apoptotic signaling pathways is of interest and requires further investigation (35,36).

Studies have demonstrated that lidocaine and other local anesthetics are capable of inducing apoptosis in neuronal and nonneuronal cells, and that apoptosis is capable of inducing necrosis (6). Thus, understanding the mechanism of lidocaine-induced apoptosis is key to finding the resolution to this problem. Werdehausen *et al* (6) reported that the BCL-2 protein overexpression or lack of caspase-9 expression abolishes apoptosis, indicating that the intrinsic mitochondrial death pathway is involved in lidocaine-induced apoptosis. Once the mechanism has been fully elucidated, an effective therapy may be identified. In the present study, it was observed that the ginsenoside Rg1 inhibits lidocaine-induced apoptosis, and this provides a novel insight for the treatment of lidocaine-induced transient neurological symptoms and cauda equina syndrome following spinal anesthesia.

In conclusion, the present study demonstrated that Rg1 provides protection against lidocaine-induced apoptosis in cultured Jurkat cells. It was shown that Rg1 is a notable antiapoptotic molecule that is capable of blocking the caspase-dependent signaling cascade in Jurkat cells. The mechanistic aspects of this study suggest that the protective effect of Rg1 on lidocaine-induced apoptosis is mediated by altering the level of BCL-2 family proteins and downregulating caspase-3 expression. This *in vitro* study provides the basis for understanding and evaluating the effect of Rg1 in the *in vivo* treatment of lidocaine-induced transient neurological symptoms and cauda equina syndrome.

## Figures and Tables

**Table I:** Primers used for quantitative polymerase chaine reaction.

Gene	Forward primer (5′ to 3′)	Reverse primer (5′ to 3′)
Caspase-3	TTCATTATTCAGGCCTGCCGAGG	TTCTGACAGGCCATGTCATCCTCA
BCL-2	CATGCCAAGAGGGAAACACCAGAA	GTGCTTTGCATTCTTGGATGAGGG

BCL-2, B-cell lymphoma-2.
